# Supplementary material for: OsWRKY5 Promotes Rice Leaf Senescence via Senescence-Associated NAC and Abscisic Acid Biosynthesis Pathway
Source: Int J Mol Sci. 2019 Sep 9;20(18):4437. doi: 10.3390/ijms20184437 (PMC6770167; doi:10.3390/ijms20184437)
Supplement: Supplementary file 1 [file ijms-20-04437-s001.pdf]

# Supplementary information

| | | Subgroups[a] | Similarity (%)[b] |
|---|---|---|---|
| OsWRKY5 | WRKYGQKMAKGNPCPRAYYRCTMASQCPVRKQVQRCAEDKSILITTYEGTHSH | | |
| AtWRKY18 | WRKYGQKVTRDNPSPRAYFRCSFAPSCPVKKKVQRSAEDPSLLVATYEGTHNH | IIa | 35/53 (66) |
| AtWRKY40 | WRKYGQKVTRDNPSPRAYFKCACAPSCSVKKKVQRSVEDQSVLVATYEGEHNH | IIa | 31/53 (58) |
| AtWRKY6 | WRKYGQKMAKGNPCPRAYYRCTMATGCPVRKQVQRCAEDRSILITTYEGNHNH | IIb | 48/53 (91) |
| AtWRKY47 | WRKYGQKMAKGNPCPRAYYRCTMAVGCPVRKQVQRCAEDTTILTTTYEGNHNH | IIb | 46/53 (87) |
| AtWRKY8 | WRKYGQKAVKNSPYPRSYYRCTTQ-KCNVKKRVERSYQDPTVVITTYESQHNH | IIc | 29/53 (55) |
| AtWRKY49 | WRKYGQKSIKNSPNPRSYYKCTNP-ICNAKKQVERSIDESNTYIITYEGFHFH | IIc | 27/53 (51) |
| AtWRKY7 | WRKYGQKPIKGSPHPRGYYKCSSVRGCPARKHVERALDDAMMLIVTYEGDHNH | IId | 30/53 (57) |
| AtWRKY17 | WRKYGQKPIKGSPHPRGYYKCSTFRGCPARKHVERALDDSTMLIVTYEGEHRH | IId | 30/53 (57) |
| AtWRKY14 | WRKYGQKPIKGSPFPRGYYRCSSSKGCSARKQVERSRTDPNMLVITYTSEHNH | IIe | 28/53 (53) |
| AtWRKY29 | WRKYGQKPIKGSPYPRSYYRCSSSKGCLARKQVERNPQNPEKFTITYTNEHNH | IIe | 26/53 (49) |
| | WRKYGQK C C H H | | |

**Fig. S1.** Amino acid sequence alignments of WRKY domains between OsWRKY5 and group II AtWRKY proteins.

Amino acid sequences of OsWRKY5 and group II AtWRKY proteins were obtained from the Rice Genome Annotation Project (http://rice.plantbiology.msu.edu/) and The Arabidopsis Information Resource (TAIR, https://www.arabidopsis.org/), respectively. Sequence alignment was performed using ClustalW with default parameters. Sequences are as follows: OsWRKY5, Os05g04640; AtWRKY18, At4g31800; AtWRKY40, At1g80840; AtWRKY6, At1g62300; AtWRKY47, At4g01720; AtWRKY8, At5g46350; AtWRKY49, At5g43290; AtWRKY7, At4g24240; AtWRKY17, At2g24570; AtWRKY14, At1g30650; AtWRKY29, At4g23550. Black boxes represent amino acids of AtWRKY proteins that are identical to those of OsWRKY5. Red and blue boxes represent the conserved WRKYGQK sequence and zinc finger motif of the WRKY domain, respectively. [a]Eulgem et al. (2000) classified group II AtWRKY proteins into five subgroups [37]. [b]Amino acid similarity of AtWRKY proteins compared with OsWRKY5. Os, *Oryza sativa*; At, *Arabidopsis thaliana*.

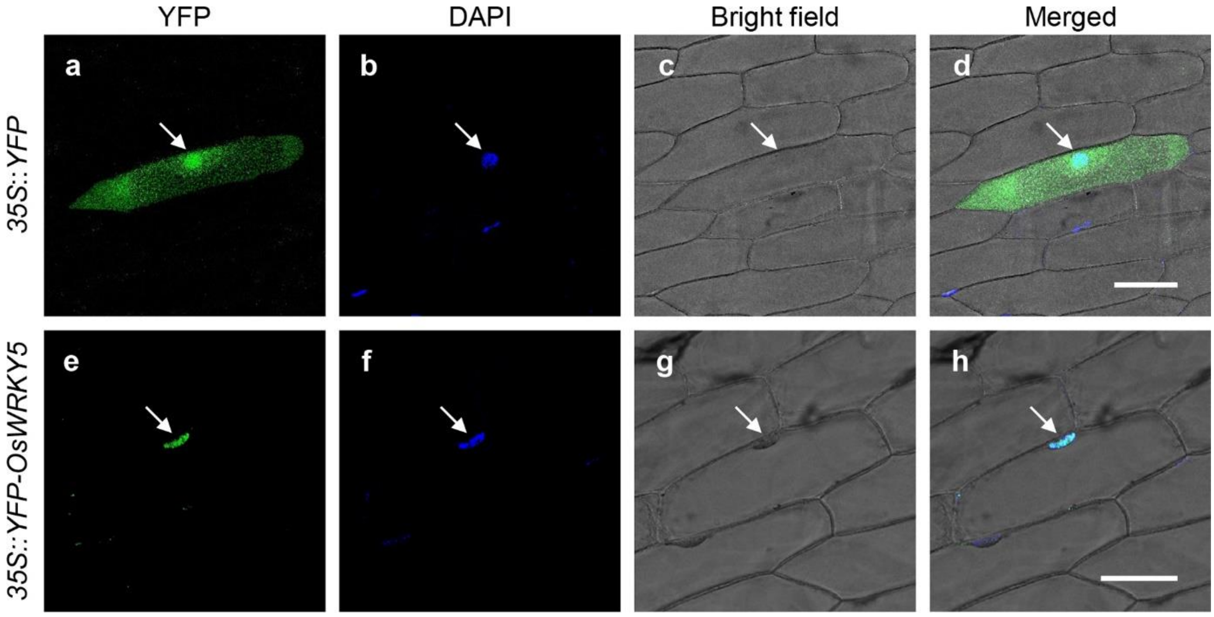


**Fig. S2.** Subcellular localization of OsWRKY5.

YFP-fused OsWRKY5 proteins transiently expressed in onion epidermal cells. Upper panels show fluorescence from the YFP control (**a-d**), which was distributed throughout the cell. Lower panels show the fluorescent signal of YFP-OsWRKY5 exclusively localized to the nucleus (**e-h**). Nuclei were stained with 4`,6-diamidino-2-phenylindole dihydrochloride (DAPI). Experiments were repeated twice with similar results. White arrows indicate the position of the nucleus. Bars = 100 μm.

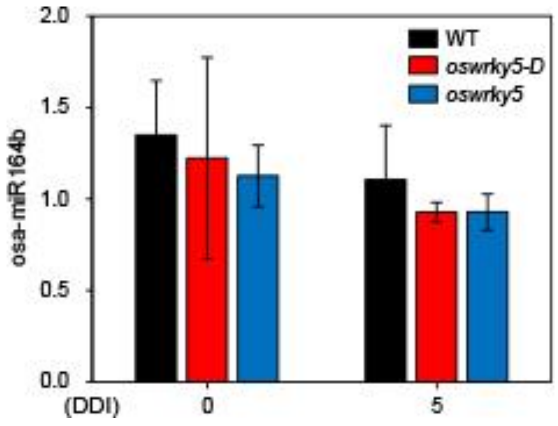


**Fig. S3.** Endogenous osa-miR164b levels in *oswrky5-D* and *oswrky5*.

Total RNA was extracted from detached leaves of WT and mutant lines (*oswrky5-D* and *oswrky5*) at 0 and 5 DDI under DIS as shown in Figure 2B. First-strand cDNA was synthesized by stem-loop pulsed RT-PCR. Transcript levels of osa-miR164b were determined by RT-qPCR and normalized using that of U6 snRNA. Mean and SD values were obtained from more than three biological replicates. No significant difference was found between WT and mutant lines by Student's *t*-test. DDI, day(s) of dark incubation.

AGAAACAACCTACCCTAGCTGGTTTTTGCACTCTGAAGTCTAAACTGAAAAGGTCTTCAGTTCTTGATATATATG
CAGATATTGTCAACTACTAGCTGATGAAGGCCTTGACTCTAGCTAAATACTAAGCCTCATGTGTCAATTTCTTCA
TGATTTCTCCACCCATATTGCGGCTGGATAATGTACACTTAGGCATCAGTCCATATGGTGTGTGAATGTGCAAGA
ACGGATCCAACACGCTAGGTAGAGTCGTAGAGATATATATAGATCCGGCCGTGAAAATGCCATCAAACCTTTCT
CATGAAACTACGCTTATGAAGAAAGAGAGTTTACCACATGTTTATCTTTTTGAATTTCATGCATATTGATTTCGAT
TGGCTACAAGTGCATGCGCATTAATCCGCAAGTGGTTGACACTGGTTTATTACTTTCTTTACATAGCAAAAAAA
AAAATAATGAAACTATGGTTGTGTTTAGTTCCCTTTAAGCTTCCAAAAATTCCGTCACATCAAATGTTTGGATAC
ATGCATAGAGCATTAAATGTGGACGAAAAAAACCAATTACACAGTTTGCATGTAAATTACGAGACGAATCTTTT
GAGCCTAATTACGCCGTGATTTGACAATGTGGTGCTACAGTAAACATTTGCTAATGGCAAATTAATTAGACTTA
ATAAATTCGTCTCACAGTTTACAGGCGGAATCTGTAATTTGTTTTATTATTAGTATATATTTAATACTTCAAATGTG
TGTCCGTATTCTTCAAAAAAAAAATTTGGAGGAGGAACTAAACACAGCCTATATTTAAAAAATGTATTTTGCAAG
ATAAATCTAATCAATATGATTAATTCTTGTGTAATTAATGATATCAAGTCAAGTAAAAGAGAGATGATTAGAAGA
AGCGAATATTAGGGAAGAACCTAATATCAGATAATTAGAAGAAGCGAGACTTTAAACTTAGATCATCTAGCAC
ACAACTTTATAGATGCTAGCCGAAAAAATCATGGACGTTTGAGAGAGAGAGAGATCGTACTGAATGGAGTTAA
TTGTTTGCTGCAGAGCGTGGCATATCCTCTCATGCACGCACCATCACTCCTAGGCTATAGCTTATCTCGATCGAT
CAACTTGGTGATCGAAAGGGAGTCTACATCGCGTTGACTCCGTTGACCCGGCCCATGCAGTACACGTGGAC
GCGAGTGCCTCATCCACCTGTCACGCTGACGTACGCCGCCGTCTGACATGGCTGGCCCACCATCCCCATCGATT
CCGATCCCCATTCTCTTGCATAATTTGGTCCAAGTCCAAGTCTTTCCGTTTCACGTTACATATGCTAGCTTCTCGT
CGTTAGCTAGCTATAGGTTAAACTACTTTACTAATTTCTCACTCTCTCTCTCTCTCTCTCTTGGAACCTAGCTAACT
AGCTAGGAGTAGTAGTAGGAGCAAGAGCCATATAAAGCTAGCTAGCTACGACCTAGCTAGCTCTCCCCTACTT
+1
TAATTGATTTCTCTCCTTCTCACTCTACTGATCGATCGATCGAGCTCTATCCATGGAGATG

**Fig. S4.** The *Cis*-elements in *OsWRKY5* promoter region.

The *cis*-elements were identified in the 1,500-bp upstream of the transcription initiation site (+1) represented by bent arrow. Red, blue, and black shaded sequences represent the W-boxes, G-box, and start codon, respectively.

**Table S1.** Primers used in this study.

| A. Primers for verification of T-DNA insertion | | |
|---|---|---|
| Primer names | Left primers (5′ → 3′) | Right primers (5′ → 3′) |
| PFG_3A-15928 | CATTAAAGCTGGACCAGATGG | AACCACTTGCGGATTAATGC |
| PFG_3A-06060 | TTGGATGCCTGATTAAGGTTG | CCGTTCTTGCACATTCACAC |
| pGA2715 | CTAGAGTCGAGAATTCAGTACA | TTGGGGTTTCTACAGGACGTAAC |
| **B. Primers for subcellular localization** | | |
| Gene | Forward primers (5′ → 3′) | Reverse primers (5′ → 3′) |
| *OsWRKY5* | ATGGAGATGATGGTGCAGAAGCA | TCAGGTGGGAGACGTGCCGCAA |
| **C. Primers for RT-qPCR** | | |
| Genes | Forward primers (5′ → 3′) | Reverse primers (5′ → 3′) |
| *OsWRKY5* | GGCTCCAATGATCAGTGATGGA | AGCCATTGTGCATCGGTAGT |
| *SGR* | AGGGGTGGTACAACAAGCTG | GCTCCTTGCGGAAGATGTAG |
| *NYC3* | TGTCGTTGCCATGTGAAGAT | TTGGTCACGCCACAAATCTA |
| *OsPAO* | GGAAATCCTAGCCAAGAAGTGTTG | CGCAGGAATCCCAGCAGTT |
| *Osh69* | CCACAACACGGATAACTT | GGTGAACACTATGGAACA |
| *Osh36* | GCACGGAGGCGAACGA | TTGAGCGGTAGCACCCATT |
| *Osl85* | GAGCAACGGCGTGGAGA | GCGGCGGTAGAGGAGATG |
| *OsNAP* | CAAGAAGCCGAACGGTTC | GTTAGAGTGGAGCAGCAT |
| *OsNAC2* | CAACTCCTGGAGAGCTGCAA | GATCTCCGGGTTCACGTCG |
| *OsNCED3* | GTGGTGCTCGACAAGGAGAA | CAGAGGTGGAAGCAGAAGCA |
| *OsNCED4* | GAGGTACGACTTCCATGGGC | TTGAGGTACGGCTTGGACAC |
| *OsNCED5* | CCCAGCTTGAAGCTTTTGCT | ACAACACTGCAACTATCCCTATCACT |
| *OsUBQ5* | ACCACTTCGACCGCCACTACT | ACGCCTAAGCCTGCTGGTT |
| **D. Primers for pulsed RT-qPCR of miR164b** | | |
| Primer names | Forward primers (5′ → 3′) | Reverse primers (5′ → 3′) |
| miR164b_pulsed RT | GTCGTATCCAGTGCAGGGTCCGAGGTATTCGCACTGGATACGACTGCACG | |
| miR164b_qPCR | GCTCTGGAGAAGCAGGGC | GTGCAGGGTCCGAGGT |

| | | |
|---|---|---|
| U6 snRNA_qPCR | CAACGGATATCTCGGCTCT | CAACTTGCGTTCAAAGACTC |
| E. Primers for yeast one-hybrid assay | | |
| Primer names | Forward primers (5′ → 3′) [a] | Reverse primers (5′ → 3′) [a] |
| OsWRKY5_GAD424_*Eco*RI and *Pst*I | GAATTCATGGAGATGATGGTGCAGAAGCAACGA | CTGCAGTCAGGTGGGAGACGTGCCGCAA |
| OsNAP-1_*Eco*RI and *Xba*I | GAATTCAGGTGTGAAAACAAATAAGA | CTCGAGAATAGTACCGCTGTGGTGAA |
| OsNAP-2_*Eco*RI and *Xba*I | GAATTCATCACGTCGTTTTTCAACTA | CTCGAGGTACCAAGGTGCTAAGATAC |
| OsNAC2-1_*Sal*I and *Xho*I | GTCGACTGTGTTCAGTTTGTCTCTTC | CTCGAGGTTAACAAGCCAGAACAAAC |
| OsNAC2-2_*Sal*I and *Xho*I | GTCGACCGGGGACATTTCAGACGTTT | CTCGAGTGGTTTTGTGGGGCTTAGAA |
| OsNAC2-3_*Sal*I and *Xho*I | GTCGACCCACTGCTATTACACAATAG | CTCGAGATCTCCCAGGAGATAAGCCA |
| OsNAC2-4_*Sal*I and *Xho*I | GTCGACTTCTCGGTTGCTGCTCGGCT- | CTCGAGGGCTAGTGATCCATCAGATC |
| OsNAC2-5_*Sal*I and *Xho*I | GTCGACCTAGCTAGTACTCCATCCGT | CTCGAGCAGGTTACTACTCCCTCCAT |

[a] The underlined nucleotides represent the restriction site for restriction enzymes.
